# Amelioration of intestinal and systemic sequelae of murine *Campylobacter jejuni* infection by probiotic VSL#3 treatment

**DOI:** 10.1186/s13099-017-0168-y

**Published:** 2017-04-11

**Authors:** Ira Ekmekciu, Ulrike Fiebiger, Kerstin Stingl, Stefan Bereswill, Markus M. Heimesaat

**Affiliations:** 1 Department of Microbiology and Hygiene, Charité-University Medicine Berlin, CC5, Campus Benjamin Franklin, FEM, Garystr. 5, 14195 Berlin, Germany; 2 Department of Biological Safety, Federal Institute for Risk Assessment (BfR), National Reference Laboratory for Campylobacter, Berlin, Germany

**Keywords:** Probiotic compound, VSL#3, Secondary abiotic mice, Gnotobiotic mice, Bacterial in vivo competition, Pathogen–commensal bacteria–host interaction, Apoptosis, Innate and adaptive immune cells, Pro-inflammatory cytokines, Anti-inflammatory cytokines, Extra-intestinal and systemic sequelae of infection

## Abstract

**Background:**

The incidence of human *Campylobacter jejuni* infections is progressively increasing worldwide. Probiotic compounds might open up valuable tools to decrease pathogen burden and subsequent pro-inflammatory immune responses, but in vivo data are scarce.

**Methods and results:**

Secondary abiotic mice generated by broad-spectrum antibiotic treatment were perorally challenged with the commercial probiotic compound VSL#3 consisting of *Streptococcus thermophilus*, *Bifidobacterium breve*, *Bifidobacterium longum*, *Bifidobacterium infantis*, *Lactobacillus acidophilus*, *Lactobacillus plantarum*, *Lactobacillus paracasei*, and *Lactobacillus delbrueckii* ssp. *bulgaricus*) either 5 days before (i.e. prophylactic regimen) or after (i.e. therapeutic regimen) peroral *C. jejuni* strain 81–176 infection, and analyzed 3 weeks following the initial bacterial re-association. Upon challenge, mice were colonized with the probiotic bacteria and/or *C. jejuni* at comparable intestinal loads, but co-colonization did not result in reduction of the pathogen burden. Remarkably, prophylactic as well as therapeutic VSL#3 treatment of *C. jejuni* infected mice ameliorated intestinal apoptosis and pro-inflammatory immune responses as indicated by lower numbers of innate and adaptive immune cell populations in the murine colon upon probiotic prophylaxis or treatment and reduced colonic concentrations of pro-inflammatory mediators including IL-6 and MCP-1. Importantly, concentrations of anti-inflammatory mediators such as IL-10 were significantly elevated in the colon of probiotics treated mice as compared to untreated controls. Strikingly, prophylactic VSL#3 treatment attenuated *C. jejuni* induced systemic pro-inflammatory responses as indicated by less TNF and IL-12p70 secretion in the spleen of VSL#3 pre-treated as compared to non-treated mice.

**Conclusion:**

Administration of probiotic formulations such as VSL#3 might open up valuable strategies for prophylaxis and/or treatment of *C. jejuni* induced intestinal and systemic sequelae in vivo by the suppression of pro-inflammatory and induction of anti-inflammatory responses.

## Background

The enteric bacterial pathogen *Campylobacter jejuni* is regarded as a commensal within the intestinal tract of wild and domestic animals, but highly virulent in humans acquiring the pathogen usually by consumption of contaminated products derived from livestock animals or contaminated surface water via the peroral route [1–3]. Whereas *Campylobacter* infections are on the rise worldwide [4–6], patients present with gastroenteritis of varying degree ranging from mild malaise and watery diarrhea to severe ulcerative colitis with inflammatory, bloody diarrhea [7]. In the vast majority of cases, intestinal disease resolves spontaneously, whereas systemic post-infectious sequelae including peripheral neuropathies such as Guillain-Barré-syndrome, Miller-Fisher syndrome or reactive arthritis might develop with a latency of weeks to months [8–10]. Due to the lack of suitable experimental in vivo models of campylobacteriosis, our understanding of the molecular mechanisms underlying *Campylobacter*-host interactions has been hampered for a long time [3, 11]. Conventionally colonized mice, for instance, are protected from *C. jejuni* infection due to the host specific microbiota composition exerting physiological colonization resistance [3, 12]. Previous results from our own experiments revealed that modification of the murine intestinal microbiota facilitated *C. jejuni* infection [12, 13]. Upon virtual eradication of the intestinal microbiota by broad-spectrum antibiotic treatment secondary abiotic mice became highly susceptible to *C. jejuni* colonization and exhibited key features of human campylobacteriosis such as apoptosis and inflammation in the colon [12]. Notably, colonization resistance was restored in secondary abiotic mice recolonized with a murine microbiota. Thus, both secondary abiotic mice and secondary abiotic animals re-colonized with a murine microbiota are well suited to unravel the triangular relationship between intestinal pathogens, bacteria and the host immune system in vivo [12, 14].

Given the importance of the distinct intestinal microbiota composition in rendering the vertebrate host resistant against enteric pathogens including *C. jejuni*, interest in the potential of “beneficial” modulations of the microbiota composition in humans as well as in livestock animals has arisen. One promising strategy is the application of probiotics, defined as live microorganisms which, when administered in adequate concentrations, bestow health benefits to the host [15]. There are numerous indications from both in vitro and in vivo studies pointing out the efficacy of probiotics in therapy and prevention of enteric infections. Strains of probiotic microorganisms such as *Lactobacillus acidophilus*, *Lactobacillus casei*, *Lactobacillus rhamnosus*, *Lactobacillus gasseri*, and *Bifidobacterium lactis*, for instance, have been shown to inhibit growth, metabolism and adhesion of enteropathogenic bacteria including *C. jejuni*, *Salmonella*, *Shigella*, enterotoxigenic *Escherichia coli* or *Vibrio cholerae* to intestinal cells [16–20]. Furthermore, effects of probiotics have been examined in clinical studies for a number of gastrointestinal diseases. For instance, randomized trials suggest that co-administration of VSL#3, a probiotic compound consisting of eight different bacterial strains [21], or *Saccharomyces boulardii* [22] significantly decrease the incidence of antibiotics associated diarrhea (AAD). Moreover, episodes of infectious diarrhea in both adults and children can be shortened by the use of probiotics [23]. A meta-analysis of 74 experimental studies, 84 clinical trials and more than 10,000 patients revealed that probiotics were effective in the therapy and prevention of several gastrointestinal diseases including AAD, *Clostridium difficile* toxin induced acute enterocolitis (the most severe form of AAD), infectious diarrhea, pouchitis and irritable bowel syndrome, but not of travelers’ diarrhea or necrotizing enterocolitis [24]. However, the underlying mechanisms of the probiotic effect are yet not fully understood. Proposed mechanisms of action include, for instance, modification of the intestinal microbiota [25], enhancement of colonization resistance [26] and intestinal barrier functions [27], as well as modulation of innate and adaptive immune functions [28].

In the present study, we examined the beneficial effects exerted by prophylactic and therapeutic treatment of *C. jejuni* infected mice with the probiotic compound VSL#3. We addressed, whether peroral VSL#3 application would lower intestinal pathogenic burden in the host, down-regulate *C. jejuni* induced pro-inflammatory sequelae and/or conversely, up-regulate anti-inflammatory immune responses not only locally (i.e. in the intestinal tract), but also in extra-intestinal compartments including systemic compartments.

## Methods

### Generation of secondary abiotic mice

Female C57BL/6j mice were bred and maintained within the same specific pathogen free (SPF) unit in the Forschungseinrichtungen für Experimentelle Medizin (FEM, Charité-University Medicine Berlin). Secondary abiotic mice virtually lacking an intestinal microbiota were generated by broad-spectrum antibiotic treatment for 8 weeks as described previously [29]. In brief, 8–10 week old mice were transferred to sterile cages and treated with a quintuple broad-spectrum antibiotic cocktail consisting of ampicillin plus sulbactam (1 g/L; Ratiopharm, Ulm, Germany), vancomycin (500 mg/L; Cell Pharm, Hannover, Germany), ciprofloxacin (200 mg/L; Bayer Vital, Leverkusen, Germany), imipenem (250 mg/L; MSD, Haar, Germany) and metronidazole (1 g/L; Fresenius, Bad Homburg, Germany) via the drinking water ad libitum for 8 weeks. Absence of cultivable bacteria in feces samples (applying thioglycolate enrichment broths; Oxoid, Wesel, Germany) for at least three consecutive weeks was used as a quality control for the successful depletion of the gut microbiota [29].

### Probiotic treatment and *C. jejuni* infection of secondary abiotic mice

Three days prior to bacterial re-colonization or infection experiments the quintuple antibiotic cocktail was withdrawn and replaced by autoclaved tap water. Mice were perorally infected with 10^9^ colony forming units (CFU) *C. jejuni* strain 81–176 in 0.3 mL sterile phosphate buffered saline (PBS) by gavage as described earlier [12]. For probiotic re-colonization, mice received a suspension of the commercial formulation VSL#3 (probiotic food supplement; Manufacturer: SIIT S.r.l, Trezzano sul Naviglio, Italy; distributed by Actial Farmaceutica, Funchal, Madeira, Portugal) consisting of the following eight bacterial species: *Streptococcus thermophilus*, *Bifidobacterium breve*, *Bifidobacterium longum*, *Bifidobacterium infantis*, *Lactobacillus acidophilus*, *Lactobacillus plantarum*, *Lactobacillus paracasei*, and *Lactobacillus delbrueckii* ssp*. bulgaricus*). A total of 4.5 × 10^11^ probiotic bacteria were dissolved in 50 mL sterile PBS. By gavaging 0.3 mL (either five days before or after *C. jejuni* infection), each mouse received 10^9^ viable probiotic bacteria as confirmed by cultural analyses of the suspensions. Mice infected either with the pathogen or re-colonized with the probiotic formulation alone as well as naive uninfected mice served as controls. Mice were continuously kept in a sterile environment (autoclaved food and drinking water) and were handled under strict aseptic conditions to prevent from contaminations.

### Sampling procedures

Tissue samples from colon, mesenteric lymph nodes (MLN) and spleen were removed under sterile conditions. Colonic ex vivo biopsies were collected in parallel for microbiological and immunological analyses. Immunohistopathological changes were determined in colonic samples that had been immediately fixed in 5% formalin and embedded in paraffin. Sections (5 μm) were stained with respective antibodies for in situ immunohistochemistry as described earlier [30].

### Quantitative analysis of *C. jejuni* or probiotic bacterial colonization

Viable *C. jejuni* strain 81–176 were detected in feces or at time of necropsy in luminal samples taken from the colon, dissolved in sterile PBS and serial dilutions cultured on Karmali- and Columbia-Agar supplemented with 5% sheep blood (Oxoid, Wesel, Germany) for two days at 37 °C under microaerobic conditions using CampyGen gas packs (Oxoid) as described earlier [12]. Probiotic bacteria of the formulation VSL#3 were quantitated in serial dilutions streaked onto Columbia-Agar supplemented with 5% sheep blood and Columbia-CNA Agar supplemented with colistin and nalidixic acid (both Oxoid) in parallel and incubated under aerobic (with 5% CO_2_), microaerophilic (in jars using CampGen gas packs; Oxoid) and obligate anaerobic (in jars using Anaerogen gas packs; Oxoid) conditions for at least 2 days. Bacterial species were identified according to their typical morphological appearances and confirmed by 16S rRNA based sequencing. The total probiotic bacterial loads in intestinal samples were assessed by the sum of identified CFU derived from the respective culture conditions. The detection limit of viable bacteria was ≈100 CFU per g.

### Immunohistochemical stainings of colonic ex vivo biopsies

In situ immunohistochemical analysis of colonic paraffin sections was performed as described previously [12, 31, 32]. Primary antibodies against cleaved caspase-3 (Asp175, Cell Signaling, USA, 1:200), Ki67 (TEC3, Dako, Glostrup, Denmark, 1:100), CD3 (#N1580, Dako, Denmark, dilution 1:10), FOXP3 (FJK-16s, eBioscience, San Diego, CA, USA, 1:100), B220 (eBioscience, 1:200) and F4/80 (# 14-4801, clone BM8, eBioscience, 1:50) were used. The average numbers of positively stained cells within at least six high power fields (HPF, 400× magnification) were determined for each animal microscopically by an independent blinded investigator.

### Cytokine detection in culture supernatants of ex vivo biopsies taken from colon, mesenteric lymph nodes and spleen

Colonic ex vivo biopsies were cut longitudinally and washed in PBS. MLN, spleen or strips of approximately 1 cm^2^ colonic tissue were placed in 24-flat-bottom well culture plates (Nunc, Wiesbaden, Germany) containing 500 μL serum-free RPMI 1640 medium (Gibco, life technologies, Paisley, UK) supplemented with penicillin (100 U/mL) and streptomycin (100 µg/mL; PAA Laboratories, Pasching, Austria). After 18 h at 37 °C, culture supernatants were tested for TNF, MCP-1, IL-6, IL-12p70, and IL-10 by the Mouse Inflammation Cytometric Bead Assay (CBA; BD Biosciences, Heidelberg, Germany) on a BD FACSCanto II flow cytometer (BD Biosciences).

### Statistical analysis

Medians and levels of significance were determined using Mann–Whitney test (GraphPad Prism v5, La Jolla, CA, USA) as indicated. Two-sided probability (p) values ≤0.05 were considered significant.

## Results

### Intestinal colonization densities in secondary abiotic mice following peroral re-colonization with probiotic bacteria and/or *C. jejuni* strain 81–176 infection

**Fig. 1:**
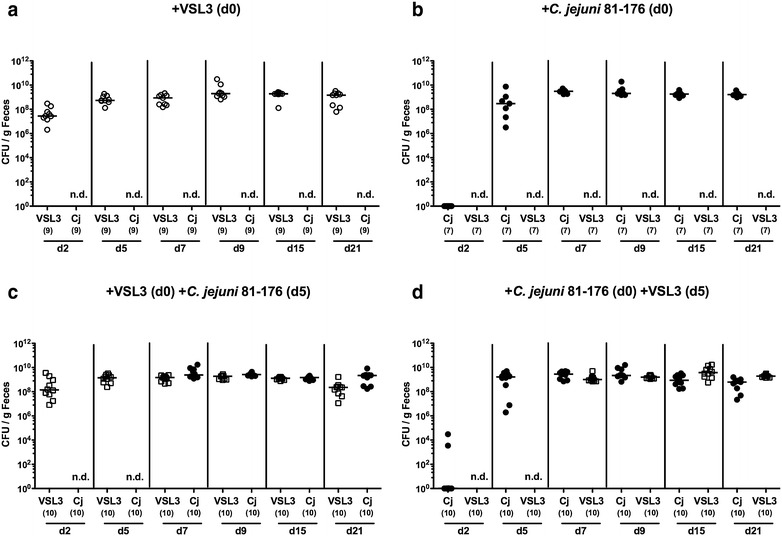
Kinetic of intestinal probiotic bacterial and/or *C. jejuni* strain 81–176 loads in perorally associated secondary abiotic mice. Secondary abiotic mice were generated by broad-spectrum antibiotic treatment and perorally re-associated with **a** the probiotic compound VSL#3 (*white symbols*) on day (d) 0, **b** *C. jejuni* strain 81–176 (Cj; *black symbols*) on d0, **c** VSL#3 on day 0 and *C. jejuni* strain 81–176 on d5 or **d** *C. jejuni* strain 81–176 (d0) and VSL#3 on d0 as described in “Methods”. Bacterial colonization densities were assessed in fecal samples (CFU/g, colony forming units per gram) over time upon re-association as indicated by culture. Medians (*black bars*) and levels of significance (p value) determined by Mann–Whitney U test are indicated. *Numbers* of analyzed mice are given in *parentheses*. Data were pooled from three independent experiments. *N.d.* not determined

**Fig. 2:**
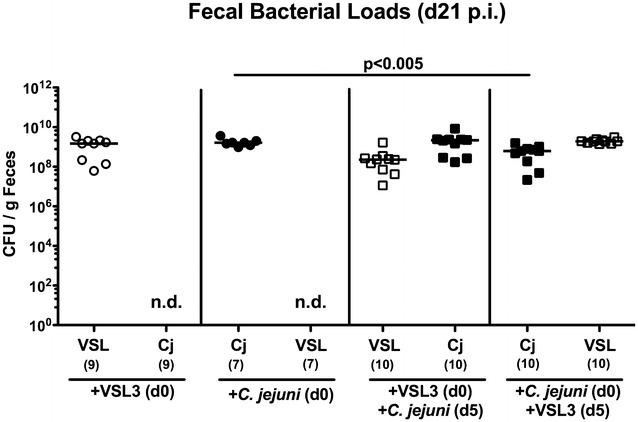
Fecal bacterial loads in *C. jejuni* strain 81–176 infected and/or VSL#3 associated secondary abiotic mice. Secondary abiotic mice were generated by broad-spectrum antibiotic treatment and perorally re-associated with the probiotic compound VSL#3 (*white symbols*) on day (d) 0, *C. jejuni* strain 81-176 (Cj; *black symbols*) on day 0, VSL#3 (day 0) and *C. jejuni* strain 81–176 (day 5) or *C. jejuni* strain 81–176 (day 0) and VSL#3 (day 5) as described in “Methods”. Bacterial colonization densities were assessed in fecal samples (CFU/g, colony forming units per gram) at day 21 upon initial re-association as indicated by culture. Medians (*black bars*) and level of significance (p value) determined by Mann–Whitney U test are indicated. *Numbers* of analyzed mice are given in *parentheses*. Data were pooled from three independent experiments. *N.d*. not determined

In the present study we investigated the potential of probiotic bacteria in the commercial formulation VSL#3 to reduce pathogen burdens and to ameliorate pro-inflammatory immune responses upon *C. jejuni* infection in vivo. To address this, secondary abiotic mice were generated by broad-spectrum antibiotic treatment of conventionally reared mice. These mice were virtually lacking intestinal bacteria and hence, physiological colonization resistance was abrogated to assure stable intestinal probiotic bacterial colonization and/or *C. jejuni* infection [11, 12]. Secondary abiotic mice were then perorally challenged with a probiotic suspension (i.e. VSL#3) containing 10^9^ viable bacteria in total by gavage either 5 days before (i.e. prophylactic regimen) or after (i.e. therapeutic regimen) peroral *C. jejuni* strain 81–176 infection (with 10^9^ CFU) and compared to control mice that were either challenged by probiotic bacteria or *C. jejuni* alone. Uninfected, naive mice served as negative controls. In fact, probiotic bacteria as well as *C. jejuni* could stably colonize the murine intestinal tract, both with high median loads of approximately 10^9^ CFU per gram feces, irrespective of the re-colonization regimen (n.s.; Fig. 1). Neither in the therapeutic nor prophylactic re-colonization group, however, probiotic bacteria were able to lower *C. jejuni* burden as indicated by comparably high pathogen loads in fecal samples over time, and the same was true the other way around (n.s.; Fig. 1c, d). If compared to *C. jejuni* mono-infected mice, however, fecal pathogen loads were approximately 0.25 orders of magnitude lower in mice of the probiotic treatment group at day 21 following *C. jejuni* infection (p < 0.005; Fig. 2).

Overall, mice could be stably re-associated with probiotic bacteria and/or *C. jejuni*, but co-colonization did not result in a biologically relevant reduction of either bacteria.

### Macroscopic and intestinal sequelae of *C. jejuni* infection and probiotic treatment

**Fig. 3:**
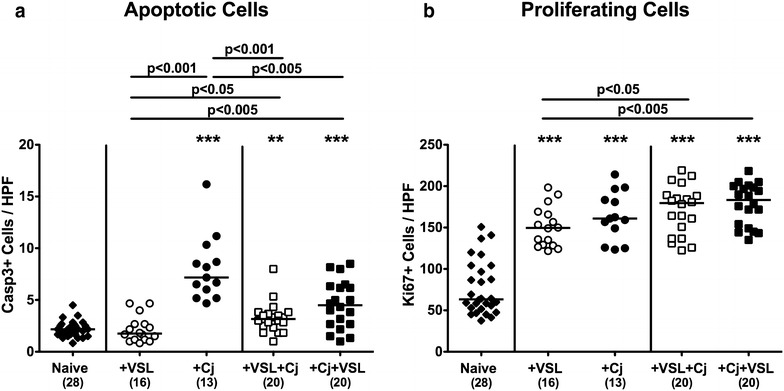
Apoptotic and proliferating cells in the colonic epithelium of *C. jejuni* strain 81–176 and/or VSL#3 associated secondary abiotic mice. Secondary abiotic mice were perorally associated either with the probiotic compound VSL#3 (+VSL; *white symbols*) or with *C. jejuni* strain 81–176 (+Cj; *black symbols*) on day 0. In bacterial competition experiments VSL#3 associated mice were challenged with *C. jejuni* strain 81–176 5 days thereafter (+VSL + Cj) or, the other way around (+Cj + VSL), *C. jejuni* infected mice additionally associated with the probiotic compound. The average numbers of colonic **a** apoptotic cells (positive for caspase-3, Casp3) and **b** proliferating cells (positive for Ki67) from six high power fields (HPF, 400× magnification) per animal were determined microscopically in immunohistochemically stained colonic paraffin sections at day 21 upon initial bacterial association. Naive mice served as uninfected controls (*black diamonds*). Medians (*black bars*), numbers of analyzed animals (in parentheses) and levels of significance (p values) determined by Mann–Whitney U test are given. Significant differences as compared to naive controls are indicated by *asterisks* (**p < 0.01; ***p < 0.001). Data were pooled from four independent experiments

Given that re-association with probiotic bacteria and/or *C. jejuni* strain 81–176 infection did not macroscopically (i.e. clinically) compromise secondary abiotic mice (not shown), we next investigated potential intestinal sequelae resulting from respective bacterial challenges. Given that apoptosis is a well-established marker for histopathological grading of intestinal inflammation and a key feature of campylobacteriosis [12], we quantitatively assessed large intestinal epithelial caspase3 + cell numbers by in situ immunohistochemistry. Numbers of colonic apoptotic cells were higher in *C. jejuni* infected mice of either group as compared to naive or VSL#3 mono-associated mice (p < 0.05–0.001; Fig. 3a). These increases, however, were far less pronounced in VSL#3 co-colonized mice of either regimen as indicated by approximately 50% lower apoptotic cell numbers as compared to *C. jejuni* infected mice (p < 0.005–0.001; Fig. 3a). Notably, re-association of mice with the probiotic compound alone was not associated with colonic apoptosis (Fig. 3a). Given that Ki67 comprises a nuclear factor necessary for cellular proliferation [33], we additionally stained colonic paraffin sections with Ki67 antibodies to assess potential proliferative (and thus regenerative) measures of the large intestinal epithelium counteracting apoptosis. Bacterial or pathogenic mono- as well as co-association resulted in increases of Ki67+ colonic epithelial cell numbers (p < 0.001; Fig. 3b) with a trend towards highest numbers in mice that were co-colonized with probiotic bacteria and *C. jejuni* (n.s. vs *C. jejuni* alone; p < 0.05–0.005 versus VSL#3 alone; Fig. 3b). Hence, prophylactic as well as therapeutic challenge of *C. jejuni* infected mice with the probiotic compound VSL#3 resulted in less pronounced large intestinal apoptotic responses.

### Intestinal and systemic pro- and anti-inflammatory responses upon probiotic treatment of *C. jejuni* infected mice

**Fig. 4:**
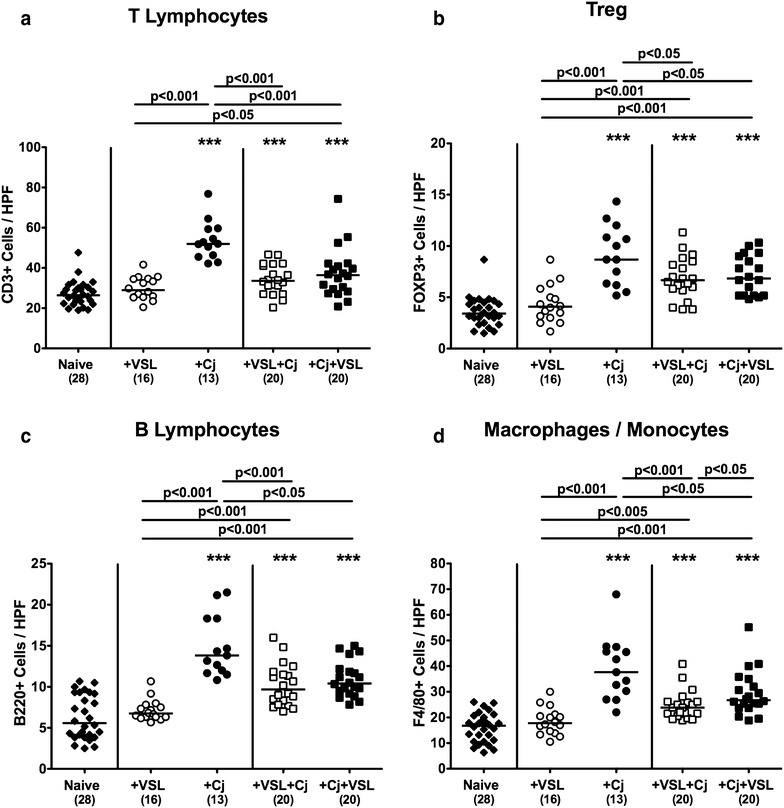
Colonic immune cell responses in *C. jejuni* strain 81–176 and/or VSL#3 associated secondary abiotic mice. Secondary abiotic mice were perorally associated either with the probiotic compound VSL#3 (+VSL; *white symbols*) or with *C. jejuni* strain 81-176 (+Cj; *black symbols*) on day 0. In bacterial competition experiments VSL#3 associated mice were challenged with *C. jejuni* strain 81–176 5 days thereafter (+VSL + Cj) or, the other way around (+Cj + VSL), *C. jejuni* infected mice additionally associated with the probiotic compound. The average numbers of colonic **a** T lymphocytes (positive for CD3), **b** regulatory T cells (Treg; positive for FOXP3), **c** B lymphocytes (positive for B220), and **d** macrophages and monocytes (positive for F4/80) from six high power fields (HPF, 400× magnification) per animal were determined microscopically in immunohistochemically stained colonic paraffin sections at day 21 upon initial bacterial association. Naive mice served as uninfected controls (*black diamonds*). Medians (*black bars*), numbers of analyzed animals (in *parentheses*) and levels of significance (p-values) determined by Mann–Whitney U test are given. Significant differences as compared to naive controls are indicated by *asterisks* (***p < 0.001). Data were pooled from four independent experiments

Since recruitment of pro-inflammatory immune cells to the site of infection is a key feature of intestinal inflammation in the course of campylobacteriosis [12], we next quantitatively assessed distinct innate as well as adaptive immune cell subsets in large intestinal ex vivo biopsies, again applying in situ immunohistochemistry. Peroral *C. jejuni* infection, but not VSL#3 re-colonization alone was associated with increases in colonic numbers of T and B lymphocytes, regulatory T cells (Treg) as well as macrophages and monocytes (p < 0.001; Fig. 4). These increases, however, were significantly less pronounced in with probiotics treated *C. jejuni* infected mice, irrespective whether VSL#3 was applied prophylactically or therapeutically (p < 0.05–0.001; Fig. 4).

**Fig. 5:**
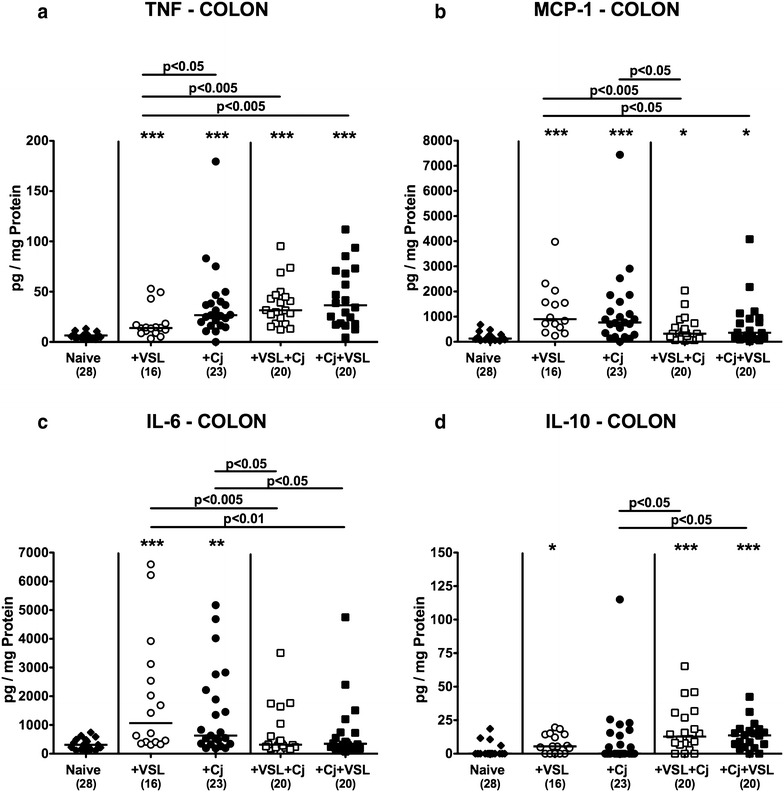
Colonic secretion of pro- and anti-inflammatory cytokines in *C. jejuni* strain 81-176 and/or VSL#3 associated secondary abiotic mice. Secondary abiotic mice were perorally associated either with the probiotic compound VSL#3 (+VSL; *white symbols*) or with *C. jejuni* strain 81–176 (+Cj; *black symbols*) on day 0. In bacterial competition experiments VSL#3 associated mice were challenged with *C. jejuni* strain 81–176 5 days thereafter (+VSL + Cj) or, the other way around (+Cj + VSL), *C. jejuni* infected mice additionally associated with the probiotic compound. **a** TNF, **b** MCP-1, **c** IL-6 and **d** IL-10 concentrations were determined in colonic ex vivo biopsies at day 21 upon initial bacterial association. Naive mice served as uninfected controls (*black diamonds*). Medians (*black bars*), numbers of analyzed animals (in *parentheses*) and levels of significance (p-values) determined by Mann–Whitney U test are given. Significant differences as compared to naive controls are indicated by *asterisks* (*p < 0.05; **p < 0.01; ***p < 0.001). Data were pooled from four independent experiments

We further measured pro- and anti-inflammatory cytokine concentrations in large intestinal ex vivo biopsies. Bacterial mono- as well as co-association were accompanied by increases in colonic pro-inflammatory mediators including TNF, MCP-1, and IL-6 (p < 0.05–0.001; Fig. 5a–c). *C. jejuni* induced increases in colonic MCP-1 and IL-6, but not increased TNF concentrations could be dampened by prophylactic probiotic treatment (p < 0.05; Fig. 5a–c). Notably, IL-6 levels were also decreased in large intestines derived from *C. jejuni* infected mice of the therapeutic VSL#3 cohort (p < 0.05; Fig. 5c) and did not differ from IL-6 concentrations measured in naive controls (n.s.; Fig. 5c). Notably, large intestinal concentrations of the anti-inflammatory cytokine IL-10 were increased upon mono- and co-association with the probiotic compound as compared to naive controls (p < 0.05–0.001; Fig. 5d). Moreover, VSL#3 application of either regimen resulted in elevated IL-10 concentrations in *C. jejuni* infected mice (p < 0.05; Fig. 5d).

**Fig. 6:**
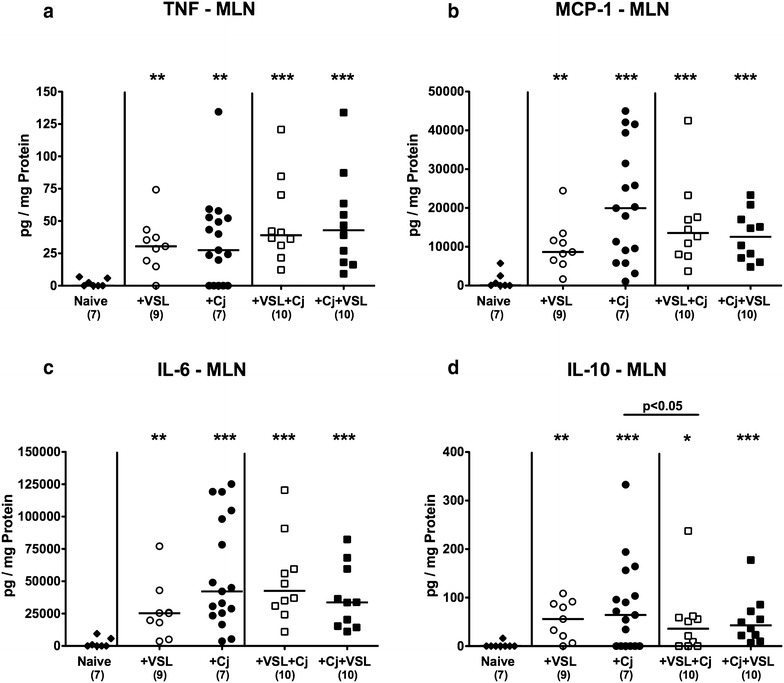
Secretion of pro- and anti-inflammatory cytokines in mesenteric lymph nodes derived from *C. jejuni* strain 81–176 and/or VSL#3 associated secondary abiotic mice. Secondary abiotic mice were perorally associated either with the probiotic compound VSL#3 (+VSL; *white symbols*) or with *C. jejuni* strain 81–176 (+Cj; *black symbols*) on day 0. In bacterial competition experiments VSL#3 associated mice were challenged with *C. jejuni* strain 81–176 5 days thereafter (+VSL + Cj) or, the other way around (+Cj + VSL), *C. jejuni* infected mice additionally associated with the probiotic compound. **a** TNF, **b** MCP-1, **c** IL-6 and **d** IL-10 concentrations were determined in ex vivo biopsies derived from mesenteric lymph nodes (MLN) at day 21 upon initial bacterial association. Naive mice served as uninfected controls (*black diamonds*). Medians (*black bars*), numbers of analyzed animals (in *parentheses*) and levels of significance (p-values) determined by Mann–Whitney U test are given. Significant differences as compared to naive controls are indicated by *asterisks* (*p < 0.05; **p < 0.01; ***p < 0.001). Data were pooled from three independent experiments

We next measured cytokine levels in another intestinal compartment. In MLN, concentrations of respective pro- and anti-inflammatory cytokines increased upon bacterial and/or pathogenic re-association (p < 0.05–0.001; Fig. 6). Prophylactic probiotic treatment of *C. jejuni* infected mice, however, resulted in slightly lower IL-10 concentrations when compared to *C. jejuni* mono-associated mice (p < 0.05; Fig. 6d).

**Fig. 7:**
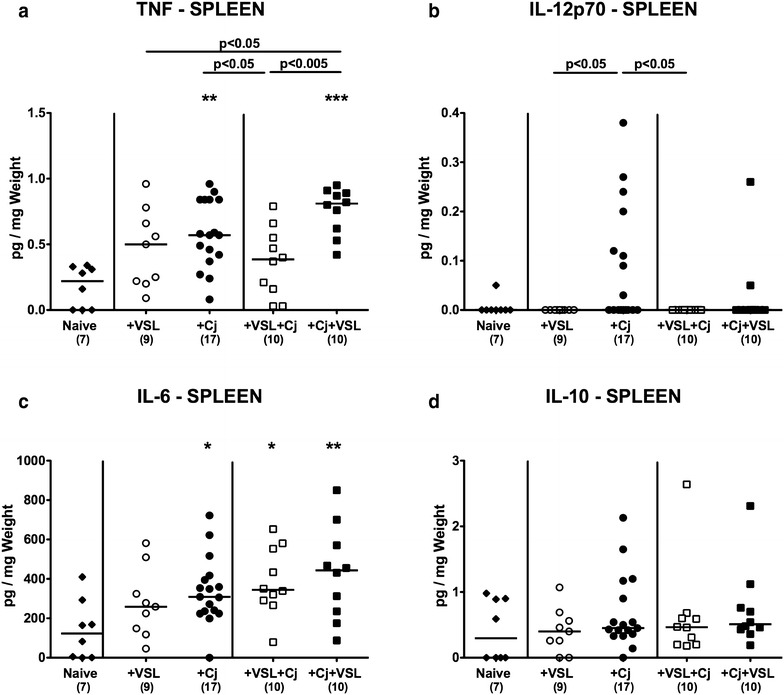
Splenic secretion of pro- and anti-inflammatory cytokines in *C. jejuni* strain 81-176 and/or VSL#3 associated secondary abiotic mice. Secondary abiotic mice were perorally associated either with the probiotic compound VSL#3 (+VSL; *white symbols*) or with *C. jejuni* strain 81-176 (+Cj; *black symbols*) on day 0. In bacterial competition experiments VSL#3 associated mice were challenged with *C. jejuni* strain 81–176 5 days thereafter (+VSL + Cj) or, the other way around (+Cj + VSL), *C. jejuni* infected mice additionally associated with the probiotic compound. **a** TNF, **b** MCP-1, **c** IL-6 and **d** IL-10 concentrations were determined in ex vivo biopsies derived from spleen at day 21 upon initial bacterial association. Naive mice served as uninfected controls (*black diamonds*). Medians (*black bars*), numbers of analyzed animals (in *parentheses*) and levels of significance (p-values) determined by Mann–Whitney U test are given. Significant differences as compared to naive controls are indicated by *asterisks* (*p < 0.05; **p < 0.01; ***p < 0.001). Data were pooled from three independent experiments

We further assessed systemic cytokine responses upon bacterial and/or pathogenic challenges of secondary abiotic mice. *C. jejuni* induced increases in splenic TNF concentrations could be slightly lowered following probiotic pre-treatment (p < 0.05; Fig. 7a), but not if probiotic treatment followed *C. jejuni* infection. In addition, mice of the prophylactic cohort exhibited lower IL-12p70 concentrations in their spleen as compared to *C. jejuni* infected animals (p < 0.05; Fig. 7b), whereas *C. jejuni* infection was associated with increased splenic IL-6 secretion (p < 0.05–0.005; Fig. 7c) that could neither be lowered by prophylactic nor therapeutic probiotic challenges. Notably, splenic IL-10 concentrations were unaffected upon bacterial re-colonization and/or pathogenic infection (n.s.; Fig. 7d).

Taken together, less colonic apoptosis upon probiotic co-administration in *C. jejuni* infected mice was accompanied by lower numbers of innate and adaptive immune cell populations in the large intestinal mucosa and lamina propria and less secretion of pro-inflammatory mediators, whereas anti-inflammatory IL-10 concentrations were increased in the colon upon prophylactic or therapeutic VSL#3 treatment of *C. jejuni* infected mice. Furthermore, prophylactic VSL#3 challenge could dampen *C. jejuni* induced TNF responses in the systemic (i.e. splenic) compartment.

## Discussion

Although *C. jejuni* are the most commonly reported bacterial etiological agents of diarrhea in developed countries [3], in vivo data regarding the molecular mechanisms underlying pathogen-host interactions are still scarce, partly due to lack of suitable mouse models mimicking *C. jejuni* induced immunopathology in humans. We have previously shown that intestinal microbial depletion following antibiotic treatment of mice can overcome physiological colonization resistance against *C. jejuni* that is elicited by the murine host specific microbiota composition [12]. Apart from that, *C. jejuni* infected secondary abiotic mice exhibit immunopathological key features of human campylobacteriosis, thus providing a well-suited model to further unravel interactions between enteropathogens and the vertebrate host [3, 11, 12]. Given the importance of the distinct microbiota composition in disease susceptibility and progression, secondary modulation of the intestinal microbiota by application of probiotic compounds has arisen as an attractive preventive or therapeutic approach. In bacterial in vivo competition experiments applying our secondary abiotic mouse model, we here investigated changes in intestinal pathogen burden and host immune responses upon peroral *C. jejuni* infection and following pre- or post-treatment with the commercially available probiotic compound VSL#3. Upon comparable and stable pathogenic as well as probiotic bacterial colonization of the intestinal tract, VSL#3 could not sufficiently decrease intestinal *C. jejuni* loads in a biologically relevant manner within 3 weeks following initial bacterial challenge. Lowering the bacterial loads in livestock animals including poultry would be of great benefit in decreasing disease transmission rates to humans via the food chain, while immunomodulatory effects such as attenuation of intestinal inflammation would additionally result in less severe disease progression in the host. Our data are in contrast to a previous study applying isolator-raised germfree BALB/c mice that had been re-associated with a complex human microbiota and treated with a probiotic mix of five different *Lactobacillus* and three *Bifidobacterium* strains [26]. Following peroral infection with *C. jejuni* the authors observed a complete eradication of *C. jejuni* from the small and large intestines of with probiotics pre-challenged “humanized” mice [26]. One needs to take into consideration, that the observed differences in pathogen-eradicative properties might be due to differences in the used probiotic mixtures and could also be explained by different immunological features of the applied animal models. Due to the lacking contact to any bacterial ligands and subsequent absence of immunological differentiation and stimulation, germfree mice exhibit only poorly-developed intestinal lymphatic tissues [34, 35]. It is thus highly reasonable that the immunological repertoire in formerly isolator raised germfree mice substantially differs from the secondary abiotic mice applied here that had been born, raised and housed under conventional conditions. In addition, reconstitution of secondary abiotic mice with eight different probiotic strains (abundant in the VSL#3 compound) might not be sufficient to reconstitute the complex physiological prerequisites for effective competition with *C. jejuni* for nutrients and niches. Instead, a well-orchestrated interplay of mucosal immunity and the intestinal intraluminal milieu determined by the concert of the complex microbiota plus beneficial probiotic strains might be required to successfully combat and/or prevent from enteropathogenic infection.

Whereas neither antibiotic treatment nor bacterial re-association compromised mice clinically, we detected more pronounced apoptotic responses in colonic epithelia following *C. jejuni* infection as reported by us previously [12, 14, 30, 36–38]. Remarkably, *C. jejuni* induced apoptosis could be alleviated by both therapeutic and prophylactic VSL#3 application. This is well in line with a former study demonstrating the capacity of VSL#3 to attenuate epithelial apoptosis in a murine dextran sodium sulphate (DSS) induced colitis model [39]. Notably, less apoptosis was associated with more than three times increased numbers of colonic epithelial Ki67+ cells as compared to naive counterparts indicative for up-regulated regenerative properties upon therapeutic as well as prophylactic VSL#3 treatment. Given that enhanced cell proliferative activity is essential in tissue repair and cell regeneration, and thus prevents from loss of epithelial integrity [40], this VSL#3 induced measure might counteract and prevent from pathogen-induced apoptosis. This mechanism has already been proposed for other probiotic species including *E. coli* Nissle 1917 [27]. The anti-apoptotic properties exerted by VSL#3 were further paralleled by a dampened *C. jejuni* induced recruitment of pro-inflammatory innate immune cell subsets including macrophages and monocytes as well as adaptive immune cell populations such as T lymphocytes, Treg and B lymphocytes into the large intestinal mucosa and lamina propria. A VSL#3 mediated attenuated influx of pro-inflammatory immune cells into the colonic mucosa has already been shown in murine trinitrobenzene sulfonic acid (TNBS) induced colitis [41]. Intestinal and extra-intestinal cytokine analyses in our study further revealed that both therapeutic and prophylactic application of the probiotic compound resulted in increased colonic secretion of the anti-inflammatory key cytokine IL-10, whereas pro-inflammatory IL-6 concentrations were decreased in large intestines as compared to untreated *C. jejuni* infected mice. In addition, VSL#3 prophylaxis resulted in decreased secretion of colonic MCP-1 upon *C. jejuni* infection. Most strikingly, anti-inflammatory properties of VSL#3 were not restricted to the intestinal tract, but could also be observed systemically, given that prophylactic VSL#3 treatment attenuated *C. jejuni* induced TNF and IL-12p70 secretion in the spleen. At the first glance it appeared somewhat confusing in this context that both mono- as well as prophylactic probiotic bacterial co-colonization resulted in elevated levels of the pro-inflammatory cytokine TNF in colon and MLN. This result was, however, further supported by a former study demonstrating that VSL#3 can in fact stimulate the intestinal epithelium to produce TNF in response, which interestingly resulted in improved epithelial barrier function and prevention of intestinal disease such as experimental ileitis [42]. Furthermore, observations that TNF deficient mice were more susceptible to acute DSS colitis have led to the concept that TNF might have protective functions in normal gut homeostasis and intestinal epithelial integrity [43]. Prophylactic VSL#3 challenge dampened *C. jejuni* induced systemic TNF responses, however. While it may be physiologically important for the host to maintain a certain well-balanced level of local inflammation as a proper response to enteropathogens, avoidance of extra-intestinal and systemic inflammatory sequelae were pivotal for host health integrity. This further supports the dichotomic properties of cytokines depending on the respective (patho-)physiological context and cytokine milieu determining whether the same cytokine acts rather pro- or anti-inflammatory [44, 45]. Our findings here further reinforce former data providing evidence for anti-inflammatory properties of VSL#3. For instance, VSL#3 has been shown to suppress MCP-1 production from human dendritic cells in vitro [46] and to down-regulate colonic MCP-1 mRNA expression also in vivo [47]. Moreover VSL#3 application could ameliorate recurrent Th1-mediated TNBS colitis in mice by inducing IL-10 and IL-10-dependent regulatory T cells expressing TGF-β [48]. The observed immunomodulatory effects of the probiotic compound might, at least in part, be attributed to the impact of probiotics on Toll-like receptor (TLR) expression [49]. We have previously reported that *C. jejuni* induced immunopathology depends on TLR-4- and TLR-9 signaling [12], whereas in a very recent study VSL#3 was shown to down-regulate TLR-2, TLR-3, TLR-4, and TLR-9 expression in vitro [50]. Whether the observed beneficial effects can be attributed to single bacterial species out of the eight probiotic strains within the compound VSL#3 or the mutualistic/synergistic interaction of distinct strains warrants further investigations.

## Conclusion

Our data presented here provide evidence that though not sufficiently enforcing pathogenic clearance, therapeutic as well as prophylactic VSL#3 application can induce anti-inflammatory responses and limit not only intestinal, but also systemic pro-inflammatory sequelae of vertebrate *C. jejuni* infection. The probiotic compound VSL#3 might therefore further open up promising tools for prophylaxis and/or treatment of *C. jejuni* induced sequelae.

## Abbreviations

AAD: antibiotics-associated diarrhea
CFU: colony forming units
DSS: dextran sodium sulphate
MLN: mesenteric lymph nodes
PBS: phosphate buffered saline
SPF: specific pathogen free
TNBS: trinitrobenzene sulfonic acid
Treg: regulatory T cells
